# Adaptation and Health: Are Countries with More Climate-Sensitive Health Sectors More Likely to Receive Adaptation Aid?

**DOI:** 10.3390/ijerph16081353

**Published:** 2019-04-15

**Authors:** Florian Weiler

**Affiliations:** Department of Social Sciences, University of Basel, 4056 Basel, Switzerland; florian.weiler@unibas.ch; Tel.: +41-61-207-1382

**Keywords:** adaptation, adaptation aid, public health, vulnerability and health, climate-sensitivity and health

## Abstract

Climate change poses a severe challenge for many developing countries, and the need to adapt has been widely recognized. Public health is one of the sectors where adaptation is necessary, as a warming climate likely affects general health conditions, the spread of various diseases, etc. Some countries are more affected by such climatic challenges, as their climate sensitivity—both to health-related issues and to climate change in general—is higher. This study examines whether more climate-sensitive countries are more likely to receive support from donors through the relatively new channel of adaptation aid, with a particular focus on the health sector. To investigate this relationship, this study proposes and operationalizes a new indicator to capture climate sensitivity of countries’ health sectors. The results, however, indicate that climate sensitivity does not matter for adaptation aid allocation. Instead, adaptation aid to a large degree follows development aid. In light of the promises repeatedly made by donors in the climate negotiations that adaptation aid should go to the most vulnerable, developing countries should push for a different allocation mechanism of adaptation aid in future negotiation rounds.

## 1. Introduction

A certain degree of climate change is no longer avoidable, and some resulting impacts such as a general warming trend, but also more local hot and cold weather extremes must therefore be expected (and are already occurring). It is crucial that countries prepare for these impacts. While climatic changes affect all countries, rich and poor, adaptation to climate impacts, ‘the process of adjustment to actual or expected climate and its effects’ [1], is of particular significance for developing countries. These countries have contributed the least to global greenhouse gas emissions, yet they are expected to be hit particularly hard by (some) climate change impacts. However, these countries often do not possess the resources necessary to cope and adapt. This injustice was already recognized in the 1992 United Nations Framework Convention to Climate Change, and developed countries agreed to assist and prioritize “particularly vulnerable” developing countries to adapt to climate change [2]. They repeatedly reconfirmed this commitment in more recent years [3,4].

Thus, adaptation finance is of particular relevance for vulnerable developing countries. Indeed, developed countries pledged in the Copenhagen Accord in 2009 to provide climate finance amounting to US$ 100 billion per year by 2020, and that there should be a ‘balance’ between mitigation and adaptation finance [3], and repeated this promise in the Paris Agreement, which also called for a significant increase of adaptation finance” [4]. While climate finance can stem from various sources, public and private [5], adaptation finance is mostly drawn from public aid budgets [6], which is why this article focuses on climate-related aid. Following the US$ 100 billion a year promise, the Organisation for Economic Cooperation and Development (OECD) Creditor Reporting System (CRS), which collects data on aid from all OECD donors, introduced separate markers to track adaptation and mitigation in 2009. Thus, each aid project reported as falling under the adaptation marker ‘intends to reduce the vulnerability of human or natural systems to the impacts of climate change and climate-related risk’ ([7], p. 4). It should be noted that questions about the reliability of these data exist [8,9,10,11], most prominently because the data are self-reported by donors and lack independent quality control, which leads to significant overestimation of aid-flows and may varies depending on the donor in question [12]. This is clearly problematic, however, some recent assessment of the data quality show that data quality has improved in recent years and ‘false positive’ reporting of projects was down to about 10% [13]. This can be considered as acceptable data quality for the purpose of this study, specifically as the occurrence of flows between donor-recipient pairs are at the center of analysis, and not the amount of aid flows identified as particularly problematic [12]. Between 2010 and 2015, the years analyzed in this study, about US$ 75 billion were tagged with the adaptation marker (as either a principal or significant contribution), about 4.8% of total development aid tracked by the OECD during that time frame. It should also be noted that the OECD includes both grants and loans to recipient countries as aid contributions, the latter if they contain concessional elements [14]. However, as less than 12% of all projects used to compile the adaptation aid were loans, this issue is not too problematic for this study.

As stated, adaptation finance is largely drawn from public (aid) budgets [6], and is to a large degree distributed via bilateral channels [8,15,16], i.e., flows directly from donor to recipient without multilateral bodies as intermediaries. Between 2010 and 2015, about 80% of the committed US$ 75 billion in adaptation funds came from bilateral donors (US$ 59.3 billion) ([8], p. 104). Because of this well-documented preference of donors for bilateral aid contributions [17], this study focuses on how these bilateral funds are disbursed. More specifically, this paper investigates the link between recipients’ vulnerability to climate change and adaptation aid allocation decision made by donors. Starting out with the promises made by donors during the climate change negotiations—and taking them at face value—there is, in principle, widespread agreement that countries ‘particularly vulnerable’ to the adverse effects of climate change should be prioritized [2,3,18,19,20]. However, a range of studies investigating this link between vulnerability and adaptation aid found mixed results, particularly studies on the subnational level, but also when focusing on multilateral funding [21,22,23,24,25,26]. On the other hand, various studies of the author of this paper and colleagues on bilateral adaptation aid instead do observe a positive link with vulnerability indicators for bilateral adaptation aid—specifically indicators related to exposure to climate change impacts and adaptive capacity [8,27,28]. However, these latter studies treat vulnerability to climate change as something effecting the entirety of countries, not individual aspects such as agriculture or public health. This study is a first attempt of disaggregating vulnerability and to investigate whether vulnerability of specific sectors influences donors’ decisions to allocate bilateral adaptation aid as well.

The interest in disaggregating vulnerability to individual sectors stems from interviews conducted with practitioners and experts in the field of climate finance conducted in Sweden, Germany, and the United Kingdom. During these interviews, respondents repeatedly talked about ‘mainstreaming of climate and adaptation finance across different sectors’ [29]. In one interview with the UK Department for International Development (DFID), the respondent pointed out that while DFID in the past had people specialized in climate change and climate adaptation, now people working on other sectors are encouraged to incorporate climate adaptation into their projects [30]. This view is also reflected in the academic literature, because it is in practice difficult to distinguish development from adaptation projects [31], or also because aiding countries to adapt to climatic impacts is equivalent to ‘good development’ [32]. In a review article, Sherman et al. found that, while there are different approaches to planned adaptation proposed in the literature, ‘all articles identified … encouraged the integration of adaptation and development’ [33]. For this article, the question then arises whether such ‘mainstreamed adaptation aid contributions’ really do consider climate vulnerability of countries in specific sectors, or whether the suggested risk of adaptation becoming no more than branding of normal development programs is closer to the truth.

The general premise of giving to the most vulnerable is equivalent to the recipient need model of the development aid literature [34]. However, while in the development aid context recipient need is commonly a measure of poverty, often simply GDP per capita [35,36,37,38,39], capturing the concept of vulnerability to climate change as recipient need for adaptation aid is more complicated [8,28]. Vulnerability is the ‘propensity or predisposition of human societies to be negatively impacted by climate hazards’ [40]. While there are various contested definitions [41], the approach which has gained wide recognition conceptualizes vulnerability as having three dimensions: physical exposure, sensitivity, and adaptive capacity [41,42,43,44,45]. ‘Exposure represents the natural and built environment that positions a system to be affected by the climate; sensitivity represents the degree a system is affected by climate stressors, and adaptive capacity is the capacity of a system to cope with such stressors’ [42]. Adger describes exposure as the ‘degree to which a system experiences environmental stress’ [41]. Consequently, the Notre Dame Global Adaptation Index (ND-GAIN) operationalizes exposure as ‘the physical factors external to the system that contribute to vulnerability’ [40]. Adaptive capacity, on the other hand, is largely a matter of resources available to combat the consequences of climate change, and thus highly dependent on countries’ GDP [8]. Thus, these two elements of vulnerability are largely external of the individual sectors affected by climate change. Sensitivity, on the other hand, does capture the degree to which a (specific) sector is affected. While past research already indicates that both physical exposure and adaptive capacity matter for adaptation aid allocation [8,28], it is the aim of this article to capture the environmental sensitivity of one specific sector and to test whether this also influences aid allocation decisions. There are various sectors potentially affected by climate change, yet due to data availability the focus of this contribution is on public health. Thus, the specific hypotheses tested in this paper is whether countries with health sectors more sensitive to climate change are more likely to receive adaptation aid. The models used in the present study are therefore an extension of those used in aforementioned work of the author of this article and colleagues [8,28]. It should be noted here that this article does not directly contribute to the literature evaluating the progress and effectiveness of adaptation in countries in general [46,47], or of individual projects [48], but only if the funds are going to countries with particularly climate-sensitive health sectors, which could then help to make progress if used effectively. 

That there is a relationship between climate change and human health has been widely recognized by a plethora of studies, and the International Panel on Climate Change (IPCC) has combined these effects into three pathways: direct impacts, effects mediated through natural systems, and effects mediated by human systems [45]. Direct effects are associations between climatic changes, mostly extreme weather events such as heat, drought, and heavy rainfall, and human health. Studies have shown that there is a link between high temperatures and the mortality rate [49,50]. On the other hand, there is also the potential that, globally, a decrease in cold spells can decrease mortality; yet the negative effects of heat extremes are expected to outweigh these effects of reduced cold spells by far [51], and especially developing countries are likely to be much more affected by increased heat than decreased cold spells [52]. Climatic effects mediated through natural systems are mostly related to various diseases, with the IPCC especially highlighting malaria, dengue fever, tick-borne diseases, and vibrio. That the prevalence of diseases is associated with temperature, and thus linked to climatic changes, has been shown for malaria [53,54], dengue fever [55,56], and tick-borne diseases [57,58]. Finally, climate change can also indirectly affect health by impacting food production, food prices, and thus the nutrition of humans. Both quality and quantity of food produced can be reduced by too high temperatures and droughts [59,60], and food prices may be driven up [45]. This can have negative consequences, for instance, on child malnutrition and stunting [61,62]. As all these effects of climate change capture the degree to which the (health) systems of developing countries are affected by external stressors, they fall into the category of sensitivity to climate change. In the section on methods below, I will outline how this sensitivity of the health sector will be operationalized, based on indicators capturing direct impacts, and indirect impacts via natural and human systems.

To assess the impact of the sensitivity of the health sector on adaptation aid allocation, the other aspects of vulnerability mentioned above must be considered as well, i.e., physical exposure as well as adaptive capacity more generally. In addition, donors also use aid to further their own commercial and strategic interests (donor interest model) [34,35,36,63], and to reward recipients for sound economic and/or democratic policies (recipient merit model) [39,64]. Thus, the models proposed here are built on models presented in earlier studies [8,28], but the focus lies on testing the impact of climate sensitivity (of one sector), which has not been done before and therefore presents the novelty of this paper.

These different aid allocation models are statistically analyzed, in combination with measures of the climate-sensitivity of the health systems, across all donors and all recipients. To conduct the research, a dyadic dataset of bilateral adaptation aid from all OECD donors between 2010 and 2015 based on project-level aid data from the OECD Creditor Reporting System was compiled. In this dataset, there is an entry for each year and each donor-recipient pair whether there was an adaptation aid flow with principal adaptation objective, i.e., projects where adaptation is the main objective. The focus on principal aid objectives stems from some problems of over-reporting related to some of the used OECD data, particularly when adaptation is only a secondary objective [10,65]. The dependent variable of the study is therefore binary in nature. The (multilevel) logit models used in this paper to analyze these aid flows allow for an understanding of the likelihood of countries to receive adaptation aid when various public health indicators—used to capture the sensitivity of health sectors—and other factors vary across countries and time. They therefore capture whether adaptation and development (here health) have been integrated, i.e., whether mainstreaming occurred.

## 2. Materials and Methods

This section briefly outlines all elements necessary for interpreting and replicating the results reported below. As already mentioned, the independent variable in all models, i.e., whether a recipient country received principal adaptation aid from a donor in a given year, comes from the OECD Creditor Reporting System aid database [66]. All bilateral aid flows between 2010 and 2015 were downloaded, then the Rio Marker for adaptation [7] was used to identify principal adaptation aid flows. When no adaptation aid between a donor and a recipient country was recorded, the adaptation aid flow for the year in question was coded as zero, and as one if such flows were reported. Overall, 23,406 entries were coded over the six-year time horizon of the study in this manner.

Overall, there are 28 donor and 141 recipient countries in the dataset, and data on adaptation aid for five years from 2010 through 2015. Of all the donors, 26 are in the dataset for the entire time period of the study, while two donors (the Czech Republic and Iceland) only started to provide adaptation aid in 2011. Thus, not only the Annex II parties required to provide funds have been selected, but all countries who provided funds for at least five years over the time-horizon of the study. However, the results of the analysis when including only Annex II parties are almost identical to those reported below. However, due to missing values on many of the covariates, described in more detail below, the number of observations in the various models is somewhat lower than that figure. Table 1 provides summary statistics for all variables (except the dummy variables LDCs, SIDS, African countries, and colonial ties).

The dependent variable thus captures the binary decision whether recipient countries receive adaptation aid or not. This binary dependent variable is then modeled using multilevel logit models, i.e., donor random effects are added to the models, as a donor’s adaptation aid allocations to various recipients in a given year cannot be regarded as entirely independent decisions. In addition, year fixed effects to capture annual fluctuations in these early years of adaptation aid flows are added to the models. The statistical models are implemented in R using the lme4 package [67]. The formula for the models used thus is:

logit (π_ij_) = β_0_ + β_1_ x_1ij_ +⋯+ β_15_ x_15ij_ + u_j_ π_ij_ = P (y_ij_ = 1|x_1ij_,…,x_15ij_,u_j_) u_j_ ~ N (0, σ_u_^2^) (1)

where y_ij_ represents the binary decision by donor j to allocate adaptation aid (1) or not (0) to recipient i, x_1ij_ to x_15ij_ are the 15 independent variables (described below) observed for observation i on which donors j base their decision, and u_j_ are the donor random effects.

In what follows the central independent variables, i.e., those measures related to public health and the health care systems of recipient countries, are introduced. In order to operationalize the proposed hypothesis, i.e., that countries with more climate sensitive health sectors are more likely to receive adaptation aid, indicators are needed which capture this sensitivity of human health and health care sectors of developing countries. Since no such measure is available, I construct a variable capturing the climate sensitivity of health systems based on three above mentioned pathways from the IPCC (direct impacts, effects mediated through natural systems, and effects mediated by human systems). For each of them three pathways, I use various indicators to construct the overall index. All indicators are taken from the Global Burden of Disease (GBD) Project, provided by the Institute for Health Metrics and Evaluation (IHME) [68], and measure the health impact in Disability-adjusted life years (DALYs). The World Health Organization (WHO) defines one DALY as one lost year of healthy life, which is the ‘sum of the Years of Life Lost (YLLs) from [a specific] cause and the Years Lost due to Disability (YLDs) for people living in states of less than good health resulting from the specific cause’ [69]. First, direct impacts of climatic changes on health, according to the IPCC, are mostly related to heat- and cold-related impacts as well as floods and storms. I use the indicator *environmental heat and cold exposure* to operationalize the former, and *risk of drowning* (for lack of a better indicator) for the latter. Second, the indicators used to capture effects mediated through natural systems, directly related to the IPCC factors mentioned in this pathway, are *malaria* and *dengue fever*, while the *prevalence of diarrhea* tries to capture more indirectly the food- and water-borne infections mentioned by the IPCC, as no direct GBD measure is provided (tick-borne diseases are omitted due to a lack of suitable indicators). Finally, effects mediated by human systems are measured by five indicators capturing nutritional deficiencies: *protein-energy malnutrition*, *iodine deficiency*, *vitamin A deficiency*, *dietary iron deficiency*, *and other nutritional deficiencies*. 

**Figure 1:**
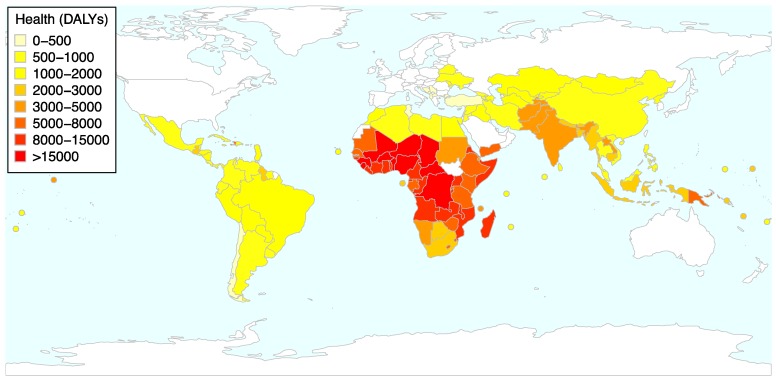
This figure shows overall impact of the indicators selected to construct the health index used as a measure to capture the sector’s climate sensitivity. The map shows the average lost DALYs over the six years included in the study lost due to the causes included in the index. Data source for all underlying variables: IHME [69].

The overall index (called GBD sensitivity index from here on) is composed for all the years included in the study (2010–2015) separately. In the models, a log-transformed version of the variable is used, as the index is heavily skewed. Figure 1 depicts the average number of DALYs lost due to the causes included in the indicator lost in the developing countries included in the study. As can be seen, the most affected countries tend to be in Sub-Saharan Africa, with a second cluster of more severely affected countries in South and Southeast Asia. This six-year averaged index ranges from only 291 DALYs in Montenegro, to a staggering 28,212 in Niger. One note of caution: as stated, this index, designed to capture sensitivity of health sectors to climatic changes, is based on health data in the years of analysis. Thus, the assumption here is that when countries experience a higher degree of climatic stress (i.e., exposure), then the countries already highly suffering from the selected causes will be the most sensitive ones. 

As a second measure of capturing the climate sensitivity of the public health sector I use the health expenditures per capita (in current US$). The idea behind this indicator is simple: the more countries spend on a per capita basis on health care, the better these countries should be prepared to the challenges faced by the health care system due to climate change. This idea, however, is very similar to the idea that countries with a higher GDP per capita are better able to deal with the consequences of climate change in general [8,28,42,70]. The more a country is able to spend, the better it is able to adapt. However, overall GDP per capita is clearly related to adaptive capacity. If GDP per capita and health expenditures are highly correlated, the danger is that the latter is simply another measure of this adaptive capacity instead of climate sensitivity. As a check, the models presented below are run once without GDP per capita, and once with the measure included, to check how the effect changes.

As just mentioned, various control variables must be included in the models in order to obtain an unbiased estimate of the just described health indicators. The first set of such control variables measure vulnerability to climate change more broadly. As already mentioned, measuring vulnerability to climate change is inherently complex. Given that climate sensitivity (of the health sector) is already taken care of by the composite health index, measure for exposure and adaptive capacity are still required [41,71].

Adaptive capacity is operationalized in two ways. First, the already mentioned financial resources assist countries with challenges posed by climate change. Second, governmental quality plays a role in how well countries are able to cope with adaptation challenges [72]. These adaptive capacity variables are measured as follows.

Countries’ financial resources are captured using per capita GDP for recipient countries, and are taken from the WDIs at 2010 constant US$ [73]. The data are lagged by one year and log-transformed. As income increases from low to high levels, a negative relationship with the likelihood of receiving adaptation aid is expected, i.e., the richer countries get the lower should be their probability of receiving aid. This variable is also an important control variable because it is highly correlated with health care spending, as already mentioned.

Governmental quality of recipient countries is measured with the World Bank’s Worldwide Governance Indicators [74]. All six main indicators (voice and accountability, political stability and absence of violence, government effectiveness, regulatory quality, rule of law, and control of corruption) are aggregated, giving equal weight to each indicator. In a second step, the obtained values are divided by six (the number of indicators in the index) in order to bring them back to the original scale of the indicators. In the statistical models the data are lagged by one year. From a donor interest perspective, higher governmental quality signals that the recipient is better able to use funds adequately, which should lead to a positive relationship with adaptation aid (i.e., the recipient merit model). From a recipient need perspective, however, lower governmental quality indicates less adaptive capacity and thus more vulnerability, which might mean governmental quality is negatively related to adaptation aid (for a much more detailed discussion of this problematic issue see [8], chapters 2.4 to 2.5). 

To capture physical exposure, a number of different concepts and indices have been proposed. However, many of these indices lump together physical exposure and adaptive capacity. In order to keep them separate, here the exposure variable provided by the Notre Dame Global Adaptation Index (ND-GAIN) is used [75].

The ND-GAIN exposure variable is particularly useful for the purpose of this paper, as it only captures country’s direct exposure to climatic changes, and is thus not connected in any way to other socio-economic variables also include in the models (such as GDP per capita as a measure of adaptive capacity, see below for more information). Thus, using this variable avoids potential collinearity problems. The variable is lagged by one year in the statistical models. Higher values indicate higher vulnerability levels, and a positive relationship with the likelihood to receive aid is therefore expected. 

In addition, three dummy variables for countries that have been recognized as “particularly vulnerable” to climate change are included in the models. These are the least developed countries (LDCs), the African countries, and Small Island Developing States (SIDS). Since these categories are not mutually exclusive, countries may belong to more than one of these groups. However, the differences between the dummies are large enough for this not to be problematic from a statistical point of view. These three country dummies are note pooled into a single variable as donors may treat the three groups of countries differently. As more vulnerable countries should obtain more adaptation aid, the coefficients of all three dummies are expected to be positive.

Next up are various measures to capture donor interests. Here a host of economic, historical, and diplomatic proxy variables are used. In contrast to vulnerability indicators that focus entirely on the recipient countries, the donor interest variables are inherently dyadic in nature. 

To capture economic interests of donors, data on bilateral trade flows are utilized, more specifically exports from the donor to the recipient country (total export of all commodities). These data are taken from the UN Comtrade database [76] and are log-transformed as well as lagged by one year. The higher the exports of donor countries to recipients, the more it is in their interest to provide aid to the partner country. Thus, a positive relationship between increased trade flows and the likelihood of adaptation aid provision is expected.

Colonial ties are also expected to play a role for (adaptation) aid allocation decisions, as donors want to sustain their influence over former colonies. As a result, former colonies are expected to be more likely to receive adaptation aid. Data on colonial ties between donors and recipients were retrieved from the Quality of Government Institute [77]. Timor-Leste was added, as it was missing in this dataset, with Portugal being the former colonial power.

Diplomatic relations and similarities in the preferences in world politics between donors and recipients are captured in the UN General Assembly Voting Data. The 2-category dyadic affinity scale ranging from −1 (least similar interests) to 1 (most similar interests) is used in this paper to capture how similar the political interests of donors and recipients are at the world stage [78]. The data are lagged by one year. The more similar the preferences of donors and recipients in the international sphere, the higher the expected likelihood that adaptation aid flows between donors and recipients are registered.

Donors have strategic interests in geographically close countries. Therefore, the (minimum) distance between a donor and a recipient country is included. This distance was calculated using the *cshapes* package of the R statistical computing environment [79]. A negative relationship between distance and adaptation aid is expected: the closer a recipient to the donor, the higher should be the likelihood of receiving adaptation aid.

In addition, the following control variables are included in the statistical models: total development aid and population. For the former, using again OECD Data [66], total bilateral development aid flows from each donor to each recipient in a given year are coded. Due to the strongly skewed nature of the data, this variable is log-transformed and lagged by one year. As the same institutions within donor countries disburse both development and climate aid, the two are expected to correlate highly. Therefore, this variable must be included the models. Population is the final control variable, and data on recipient countries’ population is taken from the WDI [73]. In the statistical models the data are lagged by one year and log-transformed. Population is included because larger countries are more likely to receive aid in general, which also holds for the adaptation aid case [28]. Thus, a positive relationship between population size and the likelihood of receiving adaptation aid is expected. Table 1 provides summary statistics for all the numerical variables (including the adaptation aid dummy) described in this section.

## 3. Results

This section presents the results of the statistical models, with the focus on the health-related variables, particularly the self-constructed GBD sensitivity index, and how they affect the likelihood of developing countries of receiving adaptation aid. A discussion about how these findings for health are related to the other variables in this analysis, and more broadly to findings reported in other studies, can then be found in the next section. Table 2 shows four models, all using the binary adaptation aid variable as the dependent variable. However, in Models 1 and 3 the two health-related indices are used without controlling for GDP per capita, while Models 2 and 4 represent the same models, yet include GDP per capita.

**Figure 2:**
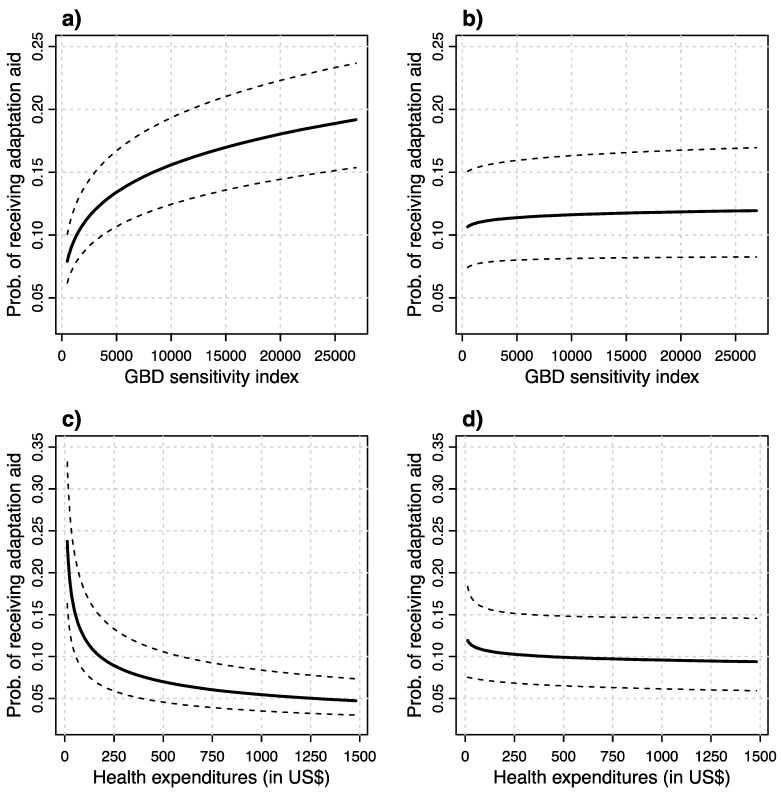
This figure shows the effects of health sensitivity to climate change on the likelihood of receiving adaptation aid. The figures in the panels (**a**) and (**b**) show the effect for the self-constructed Global Burden of Disease sensitivity index, without and with the GDP per capita control respectively. Panels (**c**) and (**d**) show the effect for health expenditures, again with and without the GDP control. The figures also show 90% confidence intervals.

As can be seen in Models 1 and 3, the health indicators seem to have the expected effect, i.e., countries scoring higher on the GBD sensitivity index are predicted to have a significantly higher likelihood of receiving adaptation aid, while countries with higher health care expenditures are predicted to receive adaptation aid less often. However, when the models include the control for GDP per capita, then both of these significant effects vanish. Figure 2 depicts the effects of the health indicators in the four models graphically. While panel (a) of the figure shows that the predicted values or receiving adaptation aid, based on Model 1 of Table 2, start at around a likelihood of 8 percent for countries with the lowest scores on the sensitivity index and increase to almost 20 percent for the most sensitive countries, panel (b) exhibits that all countries, independent of the sensitivity of their health sector to climatic changes, basically experience the same likelihood of receiving adaptation aid at slightly over 10 percent, once controlling for GPD per capita. For the health care expenditures, the results are similar. While in panel (c), showing the effect of Model 3 of Table 2, a very strong predicted effect of health care expenditures is depicted (from a likelihood of almost 25 to around 5 percent from lowest to highest expenditures), this effect again vanishes and remains relatively stable around 10 percent over the range of the health care expenditures variable once GPD per capita is included in Model 4. This is an interesting finding and shows that even health indicators that were intentionally selected to be not directly linked to per capita GDP—used to construct the GBD sensitivity index—still do have a dependency on GDP per capita and do not show significant effects once the GDP variable is included. Therefore, the self-constructed GBD index exhibits the same results as the health expenditures variable, which clearly has a strong relationship with GDP per capita. What this means for the proposed hypotheses that countries with more climate sensitive health sectors are more likely to receive adaptation aid will be discussed in the next section in detail.

## 4. Discussion

In light of the results of the health indicators presented in the last section, what can we say about donors’ consideration of recipients’ sensitivity to climate change? First of all, it should be noted that this article only looks into the climate sensitivity of a single issue, i.e., health. Of course, other potentially interesting issues which might be climate sensitive, such as agriculture or infrastructure, could show different results, and should be investigated in the future. Second, this paper also proposes a first measure to capture climate sensitivity of the health sector for all recipient countries, which necessarily relies on data currently available and has to make the assumption that countries more affected by the health impacts singled out by the IPCC will be more sensitive to future climatic changes. Thus, this measure of climate sensitivity of the health sector could also be improved by future research. However, based on the results of the present study, the proposed hypothesis that donors take into consideration the climate sensitivity of specific sectors in recipient countries—in this case the health sector—must be rejected. Thus, the mainstreaming of adaptation aid, i.e., the integration of adaptation and development, does not yet seem to have occurred. Although others find that mainstreaming does occur for development projects run directly by the EU [80], the same does not seem to be the case at the level of the individual donors, and thus for bilateral adaptation aid. This does not mean, however, that individual donors, such as for example Sweden or the UK, are not following the lead of the EU in mainstreaming climate adaptation project, but in the cross-section of 28 donor countries mainstreaming of adaptation aid cannot be detected.

**Figure 3:**
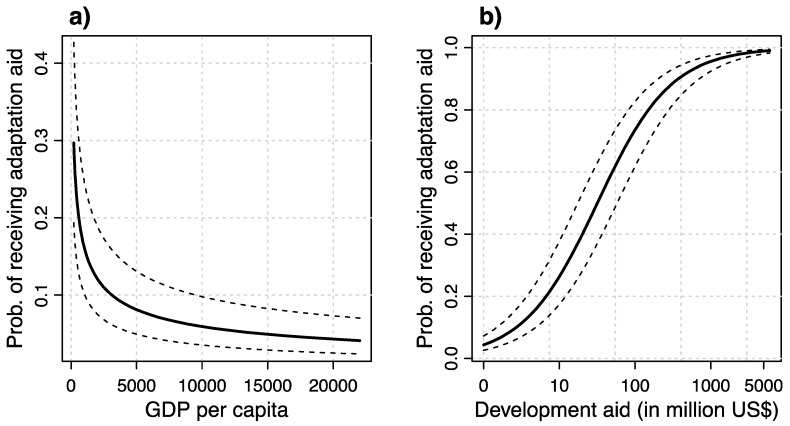
This figure shows the effects of GDP per capita (**a**) and development aid (**b**) on the likelihood of receiving adaptation aid. The figures also show 90% confidence intervals.

There is even the possibility that climate change, and vulnerability to climate change in general, is not even the main driving force behind projects tagged with the adaptation aid maker. As can be seen in Panel a) of Figure 3 (based on Model 2 of Table 2), GDP per capita is a strong predictor of the likelihood of receiving adaptation aid, with the poorest countries receiving such aid at a likelihood of almost 30 percent from a given donor. As discussed above, while per capita GDP is an indicator of adaptive capacity [8], it clearly also is a major determinant of poverty, and thus of recipient need for development aid in general [35,36,37,38,39]. In addition, Panel b) of Figure 3 shows the very strong effect spending on development aid between a given donor-recipient pair has on the latter’s likelihood of receiving aid. As can be seen, the largest recipients of development aid are also almost certain to obtain adaptation aid, and this effect is the one with the by far largest magnitude in all the models presented in Table 2. To a large degree, then, adaptation aid decisions are driven by the same allocation mechanisms as development aid projects. This idea that conventional development aid mechanisms play the major role for adaptation aid is also backed up by the findings of the donor interest variables, which show that donors care very much about their own benefit when making adaptation aid allocation decisions, as is the case for general development aid [35,36,81,82].

However, climate-related issues also do play a relatively minor, yet still significant role, as can be seen by the effect of the ND-GAIN exposure variable in all models presented in this paper. This means that of the three composites of vulnerability—exposure, sensitivity, and adaptive capacity—at least the exposure variable has some influence on adaptation aid allocation, while for adaptive capacity the role of climate vulnerability remains questionable, as the GDP per capita variable clearly captures recipient need for development aid more widely. A better measure to disaggregate the two effects would be needed. For climate-sensitivity of the health sector, the two measures used in this paper become insignificant when GDP per capita and total development aid are added as control variables. For the variable health expenditures, this should be expected, as pointed out above, as this measure correlates very highly with GPD per capita (r = 0.84), and the effect turns insignificant when both are included in the model as anticipated. However, this flushing out of the effect of the health indicator also occurs for the GBD sensitivity index, which has a much weaker correlation with per capita GDP (r = −0.40). It must therefore be concluded that climate-sensitivity does not play a role for adaptation aid allocation and that, at least for the health sector, mainstreaming of adaptation and development aid does not (yet) occur in a cross-section of donors.

## 5. Conclusions

Because there is, as yet, no systematic research on how health related climate-sensitivity influences adaptation aid allocation decisions, it is not possible to compare the health findings presented here directly to those of other studies. Nor is it possible to compare the findings presented here to any other climate-sensitive sectors, as again there is a lack of studies. Thus, more research in this area is in order, as pointed out in the previous section, for instance by designing better measures to capture the climate sensitivity of countries’ health sectors, or by investigating different sectors affected by climate change.

It is clear, however, that in the overall debate on how to allocate development aid funds health is playing an increasing role [83]. And this debate has also an explicit climate-related dimension, i.e., the sensitivity of developing countries’ health sectors to climate change, as has been shown above [45,49,50,51,52,53,54,55,56,57,58,59,60,61,62]. Mainstreaming adaptation, i.e., bringing the two dimensions of development and climate adaptation together in all aid project, is therefore desirable in general [33], and of course also when it comes to projects related to the health sector. If this mainstreaming had already occurred, adaptation aid distribution should be related, at least in part, to the climate-sensitive of countries’ health sectors. For project run by the EU itself, this mainstreaming process has already started [80], which also seems to be the case for Sweden and the UK if we believe practitioners working in these countries [29,30]. Yet, as this study shows, so far climate-sensitivity—at least of the health sector—does not play a role for how adaptation aid is allocated in a sample considering all donor-recipient pairs. Hence, the academic and policy debates on mainstreaming do not yet sufficiently spill over into adaptation aid allocation. Instead, to a large degree, adaptation aid follows the logic of development aid. As this is clearly not in line with promises made by donor countries in the UNFCCC negotiations [2,3,4], developing countries should, in the coming negotiation rounds, point towards this shortcoming in adaptation aid allocation, and demand more weight to be given to climate vulnerability, including climate sensitivity of specific sectors.

## Figures and Tables

**Table 1:** Summary statistics for all numerical variables used in the statistical models (before transformation, if used).

Variable	Mean	Median	St. Dev.	Min	Max	Valid N *
Adaptation aid (dummy)	0.23	0	0.42	0	1	23,406
GBD sensitivity index	4962	1900	6182	282	30,723	22,078
Health care expenditure (constant US$)	264.2	168.1	260.6	12.4	1192.1	22,576
Access to improved sanitation (in %)	62.3	69.8	29.1	9.1	100	21,992
Access to clean water (in %)	83.4	89.5	15.9	31.2	100	22,130
ND-GAIN exposure	0.50	0.50	0.07	0.35	0.74	22,908
GDP per capita	4040	3055	3840	213	22,366	22,380
WGI Index	−0.47	−0.47	0.63	−2.41	1.17	22,824
Eports (million US$)	555	0.2	5299	0	240,000	23,406
UN voting	0.48	0.52	0.34	−1	1	22,880
Distance (km)	6887	6599	3811	0	18,915	22,908
Population (million)	41.8	1.8	159.3	0.01	1344	22,962
Total aid (million US$)	20.4	0.15	119.3	0	6196	23,406

* The number of observations with valid N for all variables combined is 21,076. Abbreviations: GBD = Global Burden of Disease; ND-GAIN = Notre Dame Global Adaptation Initiative; WGI = Worldwide Governance Indicators; UN = United Nations; St. Dev. = Standard Deviation.

**Table 2:** Effects of health (and other) measures on the likelihood of receiving adaptation aid.

	Model 1			Model 2			Model 3			Model 4		
**Recipient need: health measures**												
GBD environmental risk (log)	0.25	***	(0.03)	0.03		(0.04)						
Health expenditure (log)							−0.38	***	(0.04)	−0.06		(0.06)
GDP per capita (log)				−0.49	***	(0.05)				−0.46	***	(0.07)
**Other vulnerability measures**												
ND-GAIN exposure	2.92	***	(0.43)	3.36	***	(0.44)	3.81	***	(0.43)	3.54	***	(0.43)
Africa (dummy)	−0.54	***	(0.06)	−0.51	***	(0.06)	−0.44	***	(0.05)	−0.50	***	(0.06)
LDCs (dummy)	0.14	**	(0.07)	−0.20	***	(0.07)	0.002		(0.07)	−0.18	**	(0.07)
SIDS (dummy)	0.05		(0.08)	0.002		(0.08)	0.01		(0.08)	−0.01		(0.08)
WGI index	0.71	***	(0.05)	0.81	***	(0.05)	0.83	***	(0.05)	0.81	***	(0.05)
**Donor interests**												
Exports (log)	0.04	***	(0.01)	0.08	***	(0.01)	0.09	***	(0.01)	0.10	***	(0.01)
Distance (log)	−0.07	*	(0.03)	−0.04		(0.04)	−0.05		(0.03)	−0.04		(0.04)
Ex-colony (dummy)	0.76	***	(0.13)	0.77	***	(0.13)	0.76	***	(0.12)	0.78	***	(0.13)
UN voting	−0.23	*	(0.12)	−0.47	**	(0.13)	−0.23	*	(0.12)	−0.43	**	(0.12)
**Other controls**												
Total aid (log)	0.92	***	(0.02)	0.89	***	(0.02)	0.89	***	(0.02)	0.89	***	(0.02)
Population (log)	0.21	***	(0.02)	0.16	***	(0.02)	0.16	***	(0.02)	0.15	***	(0.02)
Constant	−9.47	***	(0.53)	−4.14	***	(0.70)	−6.16	***	(0.56)	−3.99	***	(0.63)
Observations	21,938			21,328			22,380			22,102		
LogLikelihood	−7058			−6774			−7027			−6893		
BIC	14,307			13,747			14,265			13,986		
Groups (donors)	28			28			28			28		

Note: * *p* < 0.1, ** *p* < 0.05, *** *p* < 0.01. Yearly data lagged by one year; year dummies included (not shown).
